# Comment on Nazarloo et al. Oxytocin, Vasopressin and Stress: A Hormetic Perspective. *Curr. Issues Mol. Biol.* 2025, *47*, 632

**DOI:** 10.3390/cimb48010066

**Published:** 2026-01-07

**Authors:** Tiffany R. Lago, Federico Bolognani

**Affiliations:** Newleos Therapeutics, Inc., Boston, MA 02116, USA

Nazarloo et al. recently proposed a framework for the role of vasopressin (AVP) and oxytocin (OT) on stress hormesis, a biphasic response to low-level stressors [1]. We are inspired by the creativity of this framework and would like to add additional considerations for the role of the V1a receptor (V1aR) and its potential as a treatment target for anxiety disorders.

First, Nazarloo et al. rightly highlight the role of the vasopressin V1b receptor (V1bR) in regulating the hypothalamic–pituitary–adrenal (HPA) axis via stimulation of adrenocorticotropic hormone (ACTH) release in the anterior pituitary. However, V1aR also contributes significantly to HPA axis modulation and habituation [2], potentially through its actions within limbic structures such as the bed nucleus of the stria terminalis (BNST) [3]. Thus, both V1bR and V1aR are involved in HPA axis regulation, each operating through distinct, yet complementary, neural pathways.

Second, the authors note the importance of characterizing stressors by valence, timing, and chronicity when examining the AVP-OT system. We posit that characterizing stressors by predictability of threat may offer an even more clinically meaningful approach to understanding AVP’s mechanism of action. Responses to acute threat (“fear”) and potential threat (“anxiety”) are separate constructs. V1aR antagonists have been shown to modulate anxiety (anxiety-potentiated startle), but not fear (fear-potentiated startle), in healthy subjects [4]. Further, individuals with panic, social anxiety, specific phobias, and post-traumatic stress disorders have enhanced anxiety-potentiated startle, but not fear-potentiated startle, compared to healthy controls [5,6,7,8]. In healthy participants, selective serotonin receptor inhibitors (SSRIs) [9] and benzodiazepines [10] selectively reduce anxiety-potentiated startle without affecting fear-potentiated startle. Finally, anxiety treatments (both escitalopram and mindfulness-based stress reduction) have been shown to reduce anxiety-potentiated startle, but not fear-potentiated startle, in patients with anxiety disorders [11].

Third, we must take the opportunity to recognize that the literature presented by Nazarloo et al., in our opinion, strongly supports the therapeutic potential for V1aR antagonists in social anxiety disorder. Indeed, it is well established, as the authors note, that V1aR plays a critical role in social behavior (for review [12,13]). We add that clinical studies have demonstrated an association between a V1aR variant gene and increased amygdaloid activation during the presentation of angry and fearful faces [14]. V1aR antagonists have also been shown to decrease amygdala activation in response to threatening social cues in healthy adults [15]. In patients with autism, treatment with a V1aR antagonist has also been associated with clinically meaningful improvements in socialization and communication, as quantified by the Vineland-II Adaptive Behavior Scales [16]. Taken with the anxiolytic effects of V1aR antagonists noted above, V1aR antagonists show great promise as potential treatments for social anxiety disorder. This should be further explored in a proof-of-concept study.

We recognize the authors’ deep expertise and applaud their dedication to the neuroendocrine space. It is clear that research in the field has led to considerable advances in our understanding of the human stress response and its impressive ability to adapt. The future looks bright, and the current momentum makes now the ideal time for innovation and collaboration between academia and industry to realize the next generation of therapeutics.

## Abbreviations

The following abbreviations are used in this manuscript:

AVP	Vasopressin
OT	Oxytocin
V1aR	V1a receptor
V1bR	V1b receptor
HPA	Hypothalamic–pituitary axis
ACTH	Adrenocorticotropic hormone
SSRIs	Selective serotonin receptor inhibitors
